# Achieving NHAS 90/90/80 Objectives by 2020: An Interactive Tool Modeling Local HIV Prevalence Projections

**DOI:** 10.1371/journal.pone.0156888

**Published:** 2016-07-26

**Authors:** Jane M. Kelly, Scott D. Kelly, Pascale M. Wortley, Cherie L. Drenzek

**Affiliations:** 1 Georgia Department of Public Health, Division of Health Protection, HIV/AIDS Epidemiology, Atlanta, Georgia, United States of America; 2 Department of Mechanical Engineering and Engineering Science, University of North Carolina at Charlotte, Charlotte, United States of America; Dasman Diabetes Institute, KUWAIT

## Abstract

**Background:**

Tools using local HIV data to help jurisdictions estimate future demand for medical and support services are needed. We present an interactive prevalence projection model using data obtainable from jurisdictional HIV surveillance and publically available data.

**Methods:**

Using viral load data from Georgia’s enhanced HIV/AIDS Reporting System, state level death rates for people living with HIV and the general population, and published estimates for HIV transmission rates, we developed a model for projecting future HIV prevalence. Keeping death rates and HIV transmission rates for undiagnosed, in care/viral load >200, in care/viral load<200, and out of care (no viral load for 12 months) constant, we describe results from simulations with varying inputs projecting HIV incidence and prevalence from 2014 to 2024.

**Results:**

In this model, maintaining Georgia’s 2014 rates for diagnosis, transitions in care, viral suppression (VS), and mortality by sub-group through 2020, resulted in 85% diagnosed, 59% in care, and 44% VS among diagnosed (85%/58%/44%) with a total of 67 815 PLWH, 33 953 in care, and more than 1000 new cases per year by 2020. Neither doubling the diagnosis rate nor tripling rates of re-engaging out of care PLWH into care alone were adequate to reach 90/90/80 by 2020. We demonstrate a multicomponent scenario that achieved NHAS goals and resulted in 63 989 PLWH, 57 546 in care, and continued annual prevalence increase through 2024.

**Conclusions:**

Jurisdictions can use this HIV prevalence prediction tool, accessible at https://dph.georgia.gov/hiv-prevalence-projections to assess local capacity to meet future HIV care and social services needs. In this model, achieving 90/90/80 by 2020 in Georgia slowed but did not reverse increases in HIV prevalence, and the number of HIV-infected persons needing care and support services more than doubled. Improving the HIV care infrastructure is imperative.

## Introduction

Mathematical models have provided HIV prevalence projections under different conditions of “ramping up” HIV testing and care on a national level [1]. However, local jurisdictions vary in demographics, resource availability, health care infrastructure, undiagnosed proportions, and percent of people living with HIV (PLWH) achieving viral suppression.

Estimates of future local HIV prevalence are necessary to prepare for impending needs and for realistic goal-setting [2]. User-friendly tools incorporating local HIV data drawn from state and/or city HIV surveillance registries would help jurisdictions create tailored HIV incidence and prevalence projections to anticipate future demand for HIV care, ART, and medical and social services.

In July 2015, the White House released the new National HIV/AIDS Strategy (NHAS) 2020 goals including: 90% of PLWH diagnosed, 90% of the diagnosed in care, and 80% of the diagnosed virally suppressed (VS) [3].

We describe an approach to utilizing information available from HIV surveillance programs in the US to generate local prevalence estimates as well as the number of persons in HIV care by 2020 under varying conditions approaching the NHAS 90-90-80 target.

## Methods

### Overall approach

We created a model based on the number of persons living with diagnosed HIV, out of care with no viral load in 12 months (missing VL), in care with VL>200 (not VS), in care with VL<200 (VS) on last VL, and death rates among PLWH and the general population in Georgia in 2014, in combination with the published estimates for the percent of people with undiagnosed HIV by state, and national estimates for HIV transmission by risk behaviors. Using the NDSolve command in Mathematica 10.4, we applied rated movement from one compartment of the model to another, i.e., from undiagnosed to in care/not VS, in care/not VS to VS, VS to in care/not VS, in care/not VS to VL missing, and VS to VL missing, and varied assumptions about rates of movement. A key feature of this model is the incorporation of easily adjustable scales to create best fit simulations for varying local conditions and as new data emerges.

### Estimating the percent undiagnosed

The Centers for Disease Control and Prevention (CDC) estimates that nationally 14.0% of persons living with HIV (PLWH) are undiagnosed [4]. Using a back-calculation model based on estimated HIV incidence, adjusted for missing data, reporting delays, severity of disease at diagnosis, CDC estimates that 18.7% of PLWH in Georgia were undiagnosed in 2012 [5].

Name-based HIV reporting began in Georgia in 2004. Georgia law mandates reporting of all HIV-related laboratory test results (including undetectable viral loads) to the Department of Public Health (DPH) HIV/AIDS Epidemiology Program. Data are entered into a document-based database (enhanced HIV/AIDS Reporting System or eHARS) and laboratory results can be analyzed longitudinally.

We applied the CDC undiagnosed estimate for Georgia to the number of persons diagnosed and alive by the end of 2014 in the Georgia’s eHARS database to create an estimate of 61 404 PLWH in Georgia, of whom 11 535 were undiagnosed.

### Estimating transmission rates

Transmission of HIV depends on risk behaviors, which are known to change with awareness of HIV status, and on levels of viremia. Sexual HIV transmission rates (TR) have been estimated for those undiagnosed (TR = 0.066), in care but not on ART (TR = 0.026), in care/on ART but not VS (0.018), and in care/VS (TR = 0.004) with viral load (VL) <200 copies/ml [6]. Increasing the rate by which diagnosed persons achieved VS has been shown to lead to a reduced number of new HIV infections in San Francisco [7]. Since most jurisdictions are unable to determine which PLWH in care are on ART and not on ART, we combined these 2 categories (in care on ART/ not VS and in care/not on ART) to create an average rate for in care, not VS of 0.022.

### Dividing into subgroups based on diagnosis and viral suppression status

We subdivided the HIV positive population in Georgia according to diagnosed status and viral loads. PLWH were designated undiagnosed, in care/not virally suppressed, in care/ virally suppressed, or missing VL (no VL in 12 months) and presumed out of care. We used the symbols *u* (“undiagnosed”), *n* (“not VS”), *s*(for “suppressed”) and *m* (VL “missing”), respectively, to denote the number of PLWH in these groups.

#### Symbols and definitions

The behavior of the model depends on the number of people with undiagnosed HIV, diagnosed HIV with viral loads <200, >200, and missing in 2014, and fifteen parameters:

*τ*_*u*_ *per capita* rate of disease transmission by undiagnosed individuals
*τ*_*n*_ *per capita* rate of disease transmission by individuals in care but not virally suppressed (NVS)
*τ*_*s*_ *per capita* rate of disease transmission by individuals in care and virally suppressed (VS)
*τ*_*m*_ = *per capita* rate of disease transmission by individuals missing VL (no VL in 12 months) and presumably not in care (Missing VL)
*δ per capita* rate at which previously undiagnosed individuals are diagnosed
*σ per capita* rate of transition from in care/not VS to VS
*ρ per capita* rate at which virally suppressed individuals return to a state without viral suppression
*o*_*n*_ *per capita* rate of transition from in care/not VS to missing VL
*o*_*s*_ *per capita* rate of transition from VS to missing VL
*ϵ*_*n*_ *per capita* rate of re-entry into care from missing VL to in care/not VS
*ϵ*_*s*_ *per capita* rate of re-entry into care from missing VL to VS
*μ*_*u*_ *per capita* rate of mortality among undiagnosed individuals
*μ*_*n*_ *per capita* rate of mortality among individuals who have been diagnosed but are not virally suppressed
*μ*_*s*_ *per capita* rate of mortality among individuals who have been diagnosed and are virally suppressed
*μ*_*m*_ *per capita* rate of mortality among individuals who are missing VL

The dynamic evolution of these subpopulations can be modeled with a differential equation of the form

$$\frac{d}{dt}\;\left[\begin{array}{c} u\\ n\\ s\\ m \end{array}\right]=\;\left[\begin{array}{cccc} \tau_{u}-\delta -\;\mu_{u} & \tau_{n} & \tau_{s} & \tau_{m}\\ \delta & -\sigma -{ο}_{n}-\;\mu_{n} & \rho & \epsilon_{n}\\ 0 & \sigma & -\rho -{ο}_{s}-\;\mu_{s} & \epsilon_{s}\\ 0 & {ο}_{n} & {ο}_{s} & -\epsilon_{n}-\;\epsilon_{s}-\;\mu_{m} \end{array}\right]\left[\begin{array}{c} u\\ n\\ s\\ m \end{array}\right]$$

using the variable definitions previously described. If the quantities that appear in the square matrix above are all constants, then the differential equation is linear and can be solved analytically.

### Rates of transmission

The quantities *τ*_*u*_, *τ*_*n*_, *τ*_*y*_, and *τ*_*m*_ represent the rates at which HIV is transmitted by members of the populations *u*,*n* and *m* to previously uninfected individuals as described elsewhere [6].

We set transmission rates as fixed and assumed that

*τ*_*u*_ = 0.066
*τ*_*n*_ = 0.022
*τ*_*s*_ = 0.004
*τ*_*m*_ = 0.053

We estimated *δ* (*per capita* rate at which previously undiagnosed individuals are diagnosed) based on the estimated number of undiagnosed adults and adolescents aged >13 years in 2014 (11 535) and the number of new diagnoses among adults and adolescents made that year (2 631) or 0.23.

### Estimating death rates (*μ*_*u*_, *μ*_*n*_, *μ*_*y*_, and *μ*_*m*_)

Georgia DPH ascertains deaths by annual matching the Georgia eHARS database with Georgia Vital Records, the National Death Index and the Social Security Death Index. Because of reporting delay, we used 2010 death data. To set values for *μ*_*n*_ (death rate among those in care/not VS), *μ*_*s*_ (death rate among those diagnosed and with VS), and *μ*_*m*_ (Missing VL, presumably not in care), we counted the number of deaths in 2010 among those whose last VL in 2010 was <200 (VS) or >200 (NVS) or no VL reported in 2010 (Missing VL) (Table 1).

**Table 1:** Deaths by viral load category in 2010, Georgia.

Last VL in 2010	Total persons	Number of deaths	Rate
VS (VL<200)	13 050	153	0.0117
Not VS (VL>200)	7 664	434	0.0566
Missing VL	21 682	326	0.0150

To set a value for *μ*_*u*_, we assumed individuals living with undiagnosed HIV died from non-HIV related causes at the same rate as their age-cohort in Georgia from the Georgia Online Analytical Statistical Information System [8]. Persons diagnosed with HIV at the time of death (e.g., from an AIDS-related opportunistic infection) are included in the HIV diagnosed group. Using this rate and the estimated number of undiagnosed individuals by age group in 2012, we estimated the expected number of deaths among the undiagnosed and calculated the death rate among the undiagnosed (S1 Table).

We estimated 27 deaths/10 694 undiagnosed = 0.0025 death rate among the undiagnosed (*μ*_*u*_).

Hence, based on Georgia death data, we created mortality estimates as follows:

*μ*_*u*_ = 0.0025
*μ*_*n*_ = 0.0566
*μ*_*s*_ = 0.0117
*μ*_*m*_ = 0.015

### Estimating *σ*, *ρ*, *o*, *and ϵ*

To create a range for the rate of movement between the categories of in care/not VS (NVS) in care/VS, and out of care (missing VL) in Georgia for 2014, we used longitudinal laboratory data from eHARS documenting the transitions from viral load>200, viral load <200, and viral load missing during 2013–2014 (S2 Table).

Using these data we set the following rates as status quo conditions

### *σ* (rate of transition from NVS to VS)

$$\sigma =0.38\;(\text{NVS }2013\;->\text{ VS }2014/\text{total NVS }2013\;=\frac{2762}{7273}=0.38)$$

### *ρ* (rate of recividism, or transition from VS to NVS)

$$\rho =0.077(\text{VS }2013\;->\text{ NVS }2014/\text{total VS }2013\;=\frac{1729}{22541}=0.077)$$

### *o*_*n*_ (rate of transition from NVS to out of care [VL Missing; no VL in 12 month])

$$o_{n}=0.25(\text{NVS }2013\;->\text{ VL Missing }2014/\text{total NVS }2013\frac{1791}{7273}=0.25)$$

### *o*_*s*_(rate of transition from NVS to out of care [VL Missing; no VL in 12 month])

$$o_{s}=0.12\;(\text{VS }2013\;->\text{ VL Missing }2014/\text{total VS }2013\;=\frac{2723}{22541}=0.12)$$

### *ϵ*_*n*_ (rate of re-entry from VL MIssing to NVS)

$$\epsilon_{n}=\;0.07\;(\text{VL Missing}2013\;->\text{ NVS }2014/\text{total VL Missing }2013\;=\frac{1557}{21738}=0.07)$$

### *ϵ*_*s*_ (rate of re-entry from VL Missing to VS)

$$\epsilon_{s}=0.11\;(\text{VL Missing }2013\;->\text{ VS}2014/\text{total VL Missing }2013=\frac{2382}{21738}=0.11)$$

The number of persons with VL>200 (5693), VL<200 (22,612) and VL missing (21,617) on last viral load in 2014 for Georgia were obtained from eHARS.

## Results

**Fig 1:**
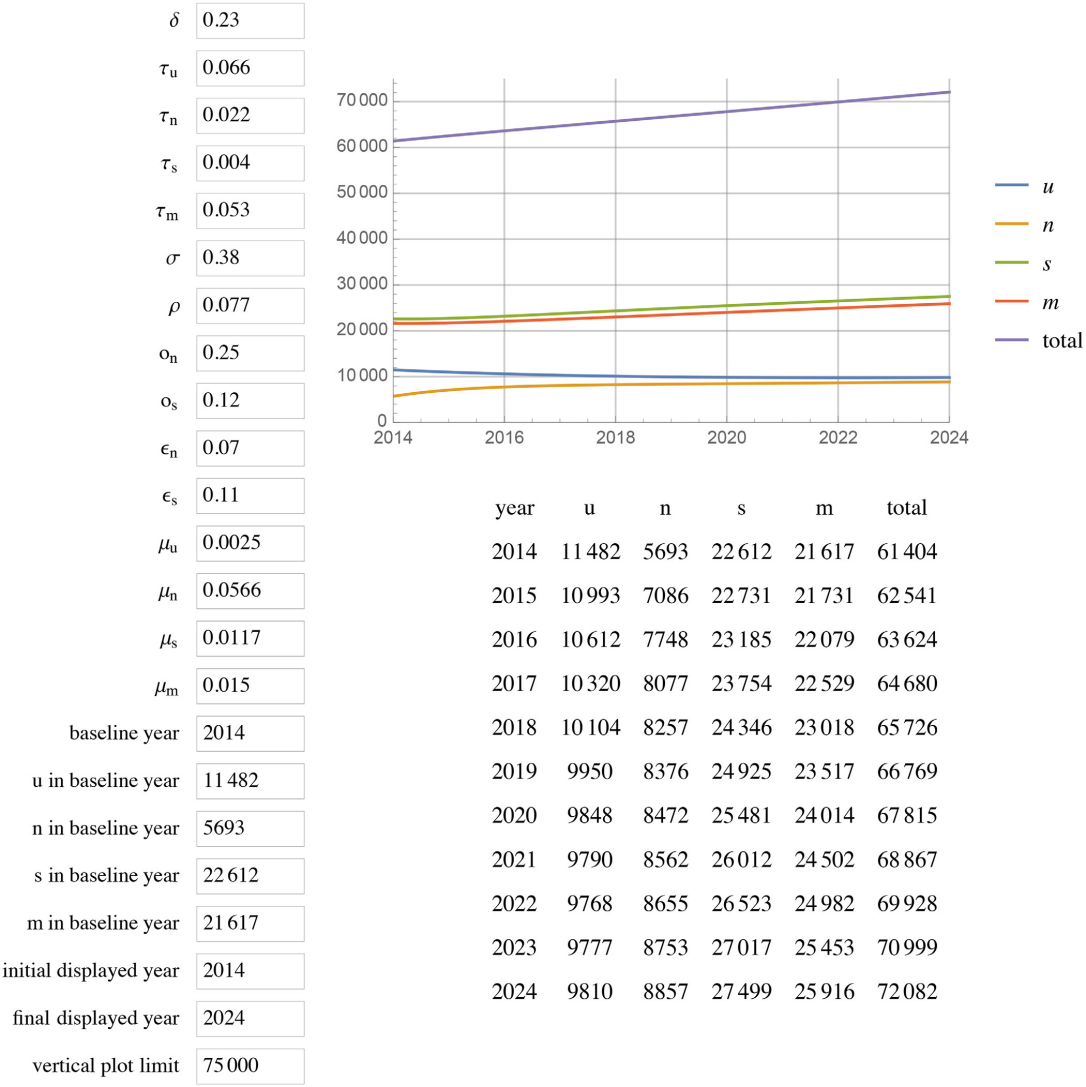
Status quo scenario. Current rates as of 2014 of diagnosis, transition between compartments, mortality, and HIV transmission estimates unchanged projected to 2024.

**Fig 2:**
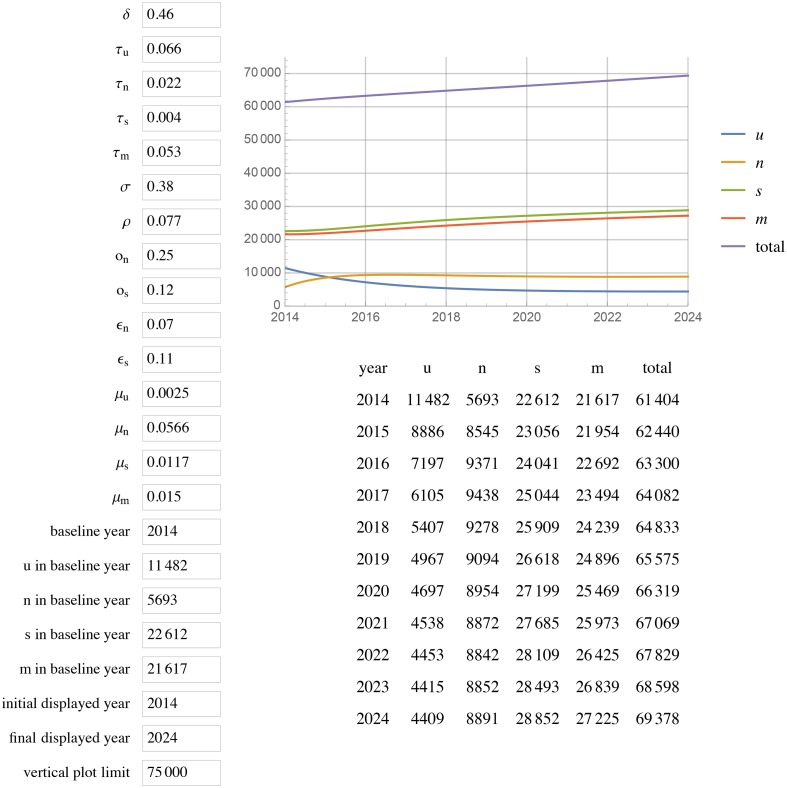
HIV prevalence projections with diagnosis rate doubled to 0.46.

**Fig 3:**
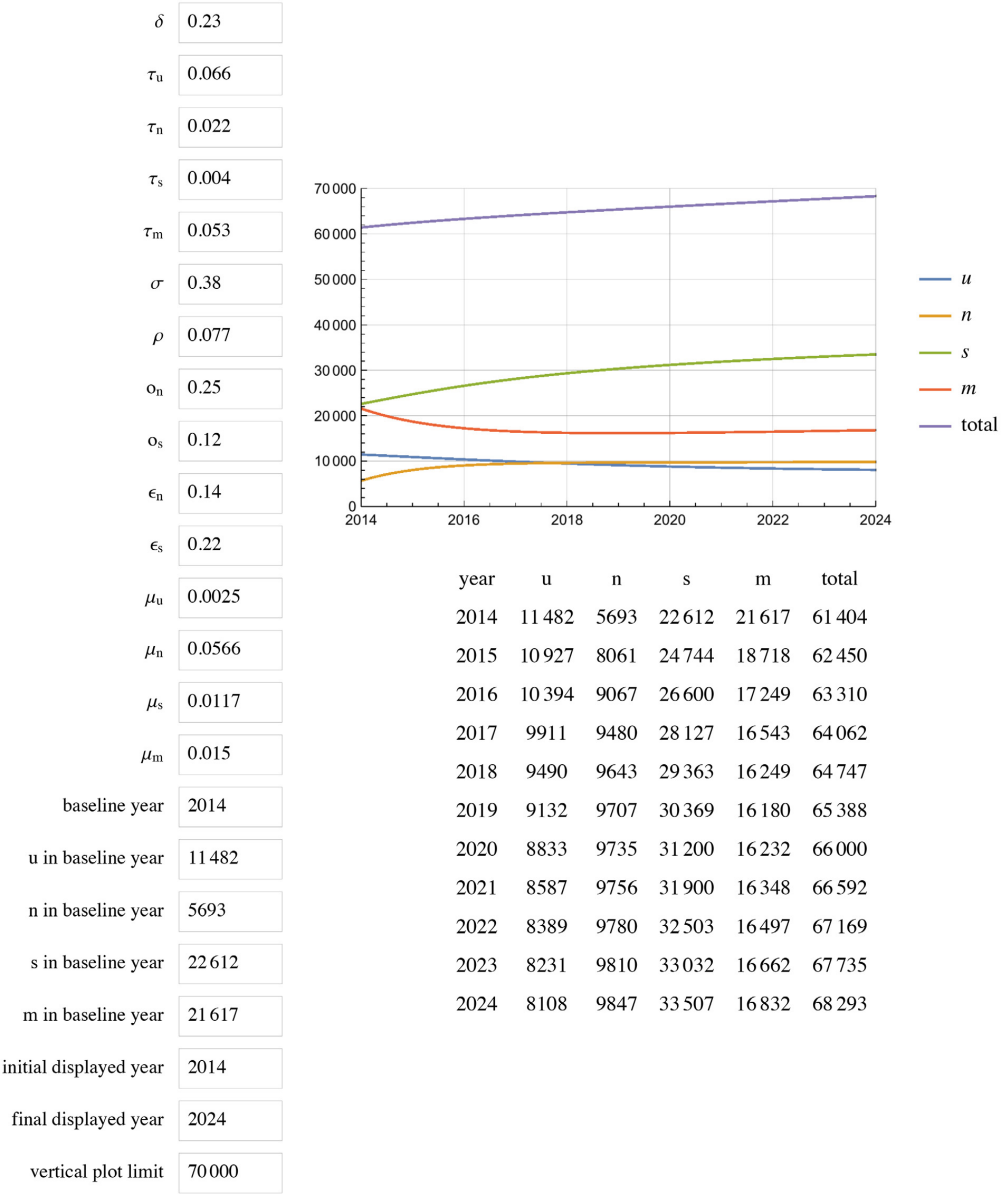
HIV prevalence projections with rates of *ϵ*_*n*_ and *ϵ*_*s*_ doubled. *ϵ*_*n*_ represents the rate of re-entry in care but not VS. *ϵ*_*s*_ represents the rate of **r**e-entry into care and achieving VS.

**Fig 4:**
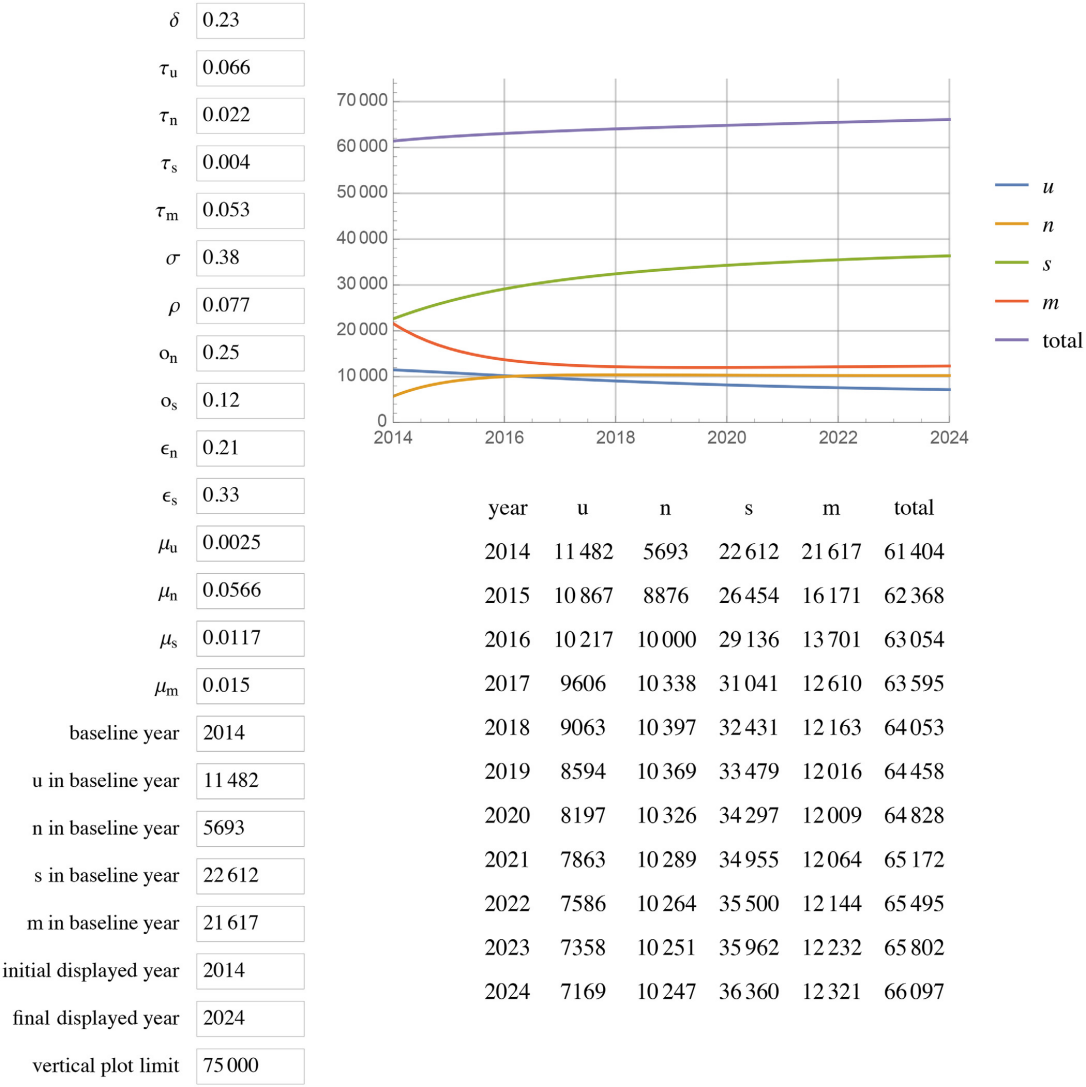
HIV prevalence projections with rates of *ϵ*_*n*_ and *ϵ*_*s*_ tripled. *ϵ*_*n*_ represents the rate of re-entry in care but not VS. *ϵ*_*s*_ represents the rate of **r**e-entry into care and achieving VS.

Fig 1 displays the results of extending this model to 2024 with unchanged parameters (status quo scenario). All projections for a given year are for year-end. In the status quo scenario, by 2020, Georgia will have reached 85% diagnosed, 59% in care among those diagnosed, and 44% VS among diagnosed (85%/58%/44%) with a total of 67 815 PLWH, 33 953 in care, and ~1000 new cases each year. Doubling the diagnosis rate increases diagnosed HIV to 93% but does not change percent in care or percent VS, with 66 139 total PLWH, 35 973 in care, and ~700 new cases each year by 2020 (Fig 2). Leaving the diagnosis rate unchanged but doubling or tripling rates of re-engaging PLWH who are out of care back into care (*ϵ*_*n*_ and *ϵ*_*s*_), results in 87%/ 72%/ 55% and 87%/79%/59% respectively (Figs 3 and 4).

As improving HIV care continuum outcomes is likely best met through multiple interventions, we modeled a scenario with multiple rate changes:

- Increase the diagnosis rate (*δ*) by 10%
- Triple the rates of re-entry into care and achieving VS (*ϵ*_*s*_) and of re-entry in care but not VS (*ϵ*_*n*_)
- Halve the rates of loss from in care but not VS to out of care (*o*_*n*_) and loss from in care and VS to out of care (*o*_*s*_)
- Double rate of transition in care but not VS to VS (*σ*)
- Halve the recividism rate of VS back to in care but not VS (*ρ*)

**Fig 5:**
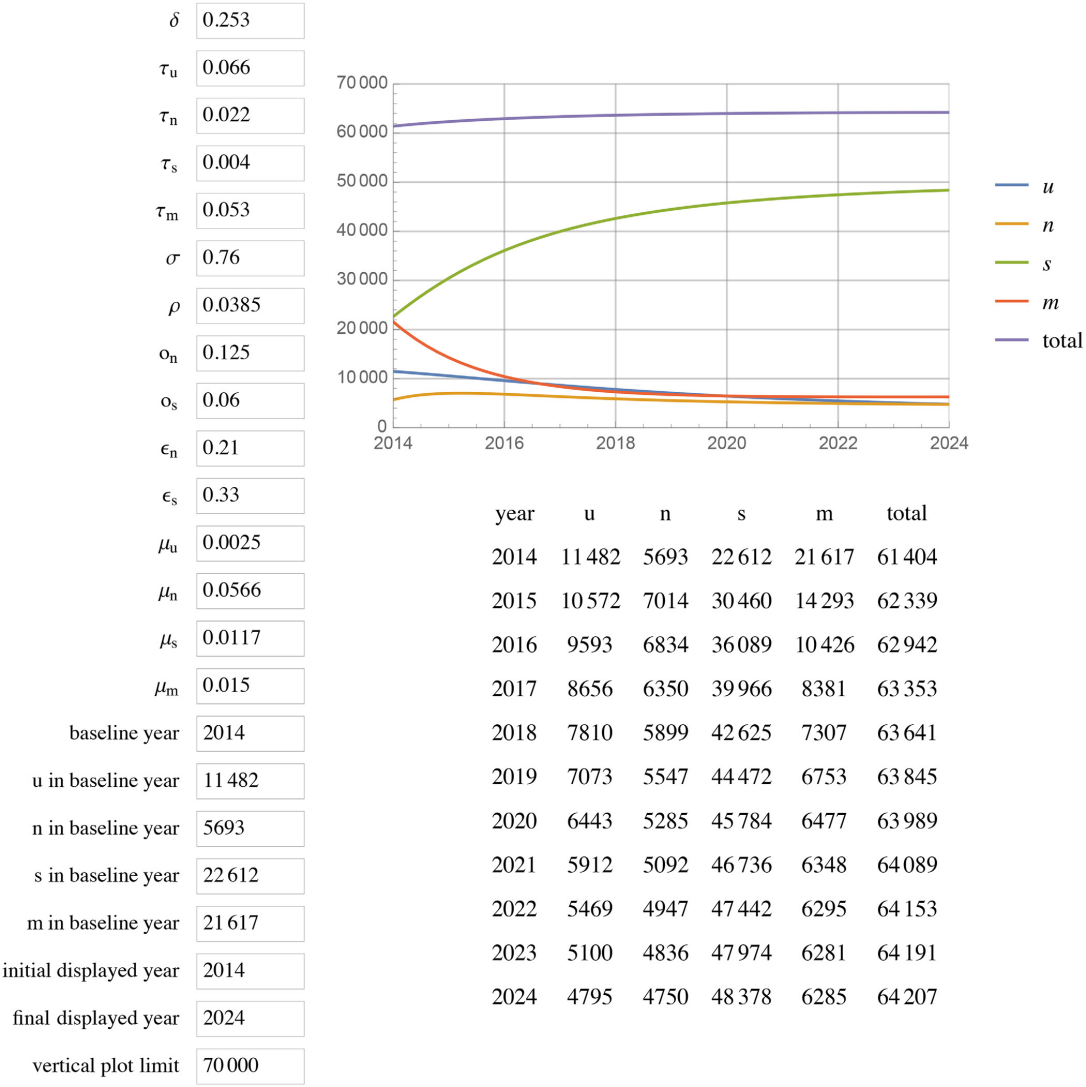
HIV prevalence projections with multiple interventions. Increase the diagnosis rate (∂) by 10%Triple the rates of re-entry into care and achieving VS (ϵ_*s*_) and of re-entry in care but not VS (ϵ_*n*_)Halve the rates of loss from in care but not VS to out of care (*O*_*n*_) and loss from in care and VS to out of care (*O*_*s*_)Double rate of transition in care but not VS to VS (*σ*)Halve the recidivism rate of VS back to in care but not VS (*ρ*)

This scenario approximates achieving the NHAS objectives by 2020 with 90% diagnosed, 89% in care among those diagnosed, and 80% VS among those diagnosed (91/89/80), with 63,989 PLWH and 57546 in care (Fig 5).

## Discussion

Meeting the NHAS goals will be much more challenging than what has been achieved thus far in HIV care and prevention. According to our model of Georgia’s HIV epidemiology, neither increasing testing alone nor tripling rates of re-engaging out of care PLWH into care were adequate to reach 90/90/80. Modeling tools, innovative strategies, and approaches tailored to local conditions are needed to increase diagnosis, re-engagement, retention, and ART adherence. Further, in this model, if Georgia were to meet 90/90/80 by 2020, HIV prevalence in Georgia would still increase through 2024 (albeit less than the status quo scenario) and the number of PLWH in care by 2020 would more than double, from 28 305 in 2014 to 57 590. Public health programs can use this model to input local HIV data and assess capacity to meet future medical and social services needs for the projected number of PLWH in their jurisdiction. In addition, the tool provides a means of visualizing the relative impact of changes in rates of diagnosis, re-engagement in care, loss to follow up, and VS. To access the interactive tool, visit https://dph.georgia.gov/hiv-prevalence-projections

Realistic goal-setting must recognize that we need be prepared for an increase in the number of HIV-infected persons needing care. Decision-makers must understand that achieving an AIDS-free generation is not the same as the end of HIV [9] nor will it mean a decrease HIV care funding needs.

There are multiple challenges to increasing HIV screening and ART coverage. Increased HIV testing would likely require implementation of routine screening in healthcare settings. However, not all state Medicaid programs reimburse for routine HIV screening [10].

Shortages in workforce capacity in HIV clinical care providers have been noted for years [11]. In 2015, only 228/327 (70%) of Infectious Disease fellowship positions were filled reflecting a downward trend from 85% in 2011 [12]. The shortage of Infectious Disease physicians in the face of an increasing HIV patient population challenges the approach of HIV care delivered predominantly by specialists, and compels us to change our healthcare model for HIV to comprehensive primary care and team care [13]. Telemedicine can increase the reach of specialists to rural areas, but the fact remains that the current health care system does not have the capacity to absorb the influx of new patients if all HIV positive clients were linked to care.

Concerns remain about whether we have the capacity to expand not only medical care but support services, e.g., mental health and substance abuse counseling, housing, and social services. PLWH have known higher levels of poverty, mental illness (both mood and thought disorder), alcohol and non-prescription drug or prescription drug abuse [14]. In addition, new holistic care models are needed for the aging cohort of PLWH who have co-morbid medical conditions and social needs associated with aging.

National HIV transmission modeling estimates that persons diagnosed but not in care account for 61.3% of HIV transmissions compared to 8.5% for those retained in care [6]. A multidisciplinary approach that includes coordination of clinical and surveillance resources to identify out-of-care persons and re-engage in care has demonstrated effectiveness [15, 16], but we need to greatly expand the clinical infrastructure in HIV care with support for long-term retention and ART adherence. Cross-sectional 12 month care continuum monitoring may be misleading: a 36 month longitudinal analysis at an urban HIV care center in Georgia found that while most of the patients met retention and VS criteria for a single year, only a minority did so over three years [17]. If we hope to reach the 2020 NHAS goals we must find ways to improve long-term continuous retention in care.

In our model, even with achievement of the NHAS 90–90–80 by 2020 targets, 27% of all PLWH (diagnosed and undiagnosed) will have a detectable viral load, with consequences for personal and public health. Additional means of reducing transmission are needed. Revision of punitive HIV criminalization laws that reinforce stigma may impact testing and treatment for key populations [18]. Harm reduction approaches such as needle exchange programs are proven effective strategies to reduce HIV transmission rates [19, 20].

Pre-exposure prophylaxis (PrEP), which is at least 92% effective in preventing HIV when taken consistently, may play a role in changing population-level transmission if used widely enough and with sufficient adherence. Unfortunately, other studies have shown decreased PrEP effectiveness with low medication constancy [21]. We need to improve PrEP access, support adherence, and reduce stigma surrounding this harm reduction intervention.

Public health policy, community advocates, and political leaders have stated support for the NHAS 90-90-80 by 2020 targets [22]. The authors’ concern is that the rhetoric of “a world free from AIDS” fails to acknowledge the need to prepare for the immediate future burden of a greatly increased number of PLWH needing services, HIV health care providers, and funding for medications. We hope this modeling tool will help local medical systems and public health prepare for this future.

## Limitations

Estimates based on HIV surveillance are subject to ascertainment bias. Missing VL values may be because the test was not done, not reported, or that the patient moved to another jurisdiction or died yet the death has not been reported, and can account for a substantial proportion of missing VL in some jurisdictions [23]. Missing VL data could lead to an overestimation of transmission since these persons are assumed to be not VS.

Transmission rates in our model were based on estimates for sexual transmission not injection drug use (IDU) because of the low proportion of IDU among PLWH in Georgia. While the proportion of persons who contracted HIV via IDU is generally small, the transmission rates are higher, and HIV outbreak events have been associated with changing patterns of IDU [24].

Death rates used in 2010 may change over time as the population of PLWH age, or more effective HIV treatments (e.g., long-acting injectable agents) are found. Death rates for persons with undiagnosed HIV may differ from that of the general population in the same age cohort, i.e., such persons are likely to have behavioral risk factors that may lead to increased non-HIV related mortality. We lack a means to quantify this. The impact of this mortality on our model is small, however.

The simulations in this model assume steady-state rates of movement from one model compartment to another whereas rates of diagnosis and viral suppression will likely change with time, or require ever-greater scale-up of effort to maintain rates, because the remaining undiagnosed and untreated individuals are the hardest-to-reach populations.

## Conclusion

A strength of the tool we have developed is that it can be easily modified with adjustable ranges to modify rates based on local data, changing epidemiology, new transmission estimates, vaccine availability, or emerging HIV treatment successes. The rising prevalence predictions demonstrated here are an imperative call to improving HIV care infrastructure.

## Supporting Information

S1 Table Estimating death rates among undiagnosed PLWH, Georgia, 2012. The death rates from the Georgia Department of Public Health Online Analytical Statistical System (OASIS) and estimated number of undiagnosed people living with HIV (PLWH) can be used to estimate the number of deaths for undiagnosed PLWH by age group.
(DOCX)

S2 Table Transitions in viral load category for PLWH in Georgia 2013–2013. Rates of movement between compartments defined by viral load (VL) measurement were calculated from Georgia eHARS data from 2013 and 2014.
(DOCX)
