# Supplementary material for: Achieving NHAS 90/90/80 Objectives by 2020: An Interactive Tool Modeling Local HIV Prevalence Projections
Source: PLoS One. 2016 Jul 26;11(7):e0156888. doi: 10.1371/journal.pone.0156888 (PMC4961282; doi:10.1371/journal.pone.0156888)
Supplement: S1 Table — The death rates from the Georgia Department of Public Health Online Analytical Statistical System (OASIS) and estimated number of undiagnosed people living with HIV (PLWH) can be used to estimate the number of deaths for undiagnosed PLWH by age group. (DOCX) [file pone.0156888.s001.docx]

**S1 Table. Estimating death rates among undiagnosed PLWH, Georgia, 2012**

| Age Group (years) | Death Rate from OASIS | Number of undiagnosed PLWH | Estimated number of deaths by age group for undiagnosed |
| --- | --- | --- | --- |
| 13-24 | 67/100,000 | 1909 | 1.3 |
| 25-34 | 110/100,000 | 2737 | 3.0 |
| 35-44 | 183/100,000 | 1946 | 3.6 |
| 45-54 | 459/100,000 | 1459 | 6.7 |
| 55 + | 2,157/100,000 | 584 | 12.6 |
