# Supplementary material for: Achieving NHAS 90/90/80 Objectives by 2020: An Interactive Tool Modeling Local HIV Prevalence Projections
Source: PLoS One. 2016 Jul 26;11(7):e0156888. doi: 10.1371/journal.pone.0156888 (PMC4961282; doi:10.1371/journal.pone.0156888)
Supplement: S2 Table — Rates of movement between compartments defined by viral load (VL) measurement were calculated from Georgia eHARS data from 2013 and 2014. (DOCX) [file pone.0156888.s002.docx]

**S2 Table Transitions in viral load category for PLWH in Georgia 2013-2014.**

|  | 2014 | | | |
| --- | --- | --- | --- | --- |
| 2013 | VS (VL<200) | Not VS (VL>=200) | VL Missing (No VL in 12 months) | Total |
| VS  (VL<200) | 18089 | 1729 | 2723 | 22541 |
| Not VS (VL>=200) | 2762 | 2720 | 1791 | 7273 |
| VL Missing (No VL in 12 months) | 2382 | 1557 | 17799 | 21738 |
| Total | 23233 | 6006 | 22313 | 48994 |
